# IMU-based human activity recognition and payload classification for low-back exoskeletons

**DOI:** 10.1038/s41598-023-28195-x

**Published:** 2023-01-21

**Authors:** Mattia Pesenti, Giovanni Invernizzi, Julie Mazzella, Marco Bocciolone, Alessandra Pedrocchi, Marta Gandolla

**Affiliations:** 1 Department of Electronics, Information and Bioengineering, Nearlab, Politecnico di Milano, 20133 Milan, Italy; 2 Department of Mechanical Engineering, Politecnico di Milano, 20156 Milan, Italy

**Keywords:** Biomedical engineering, Occupational health

## Abstract

Nowadays, work-related musculoskeletal disorders have a drastic impact on a large part of the world population. In particular, low-back pain counts as the leading cause of absence from work in the industrial sector. Robotic exoskeletons have great potential to improve industrial workers’ health and life quality. Nonetheless, current solutions are often limited by sub-optimal control systems. Due to the dynamic environment in which they are used, failure to adapt to the wearer and the task may be limiting exoskeleton adoption in occupational scenarios. In this scope, we present a deep-learning-based approach exploiting inertial sensors to provide industrial exoskeletons with human activity recognition and adaptive payload compensation. Inertial measurement units are easily wearable or embeddable in any industrial exoskeleton. We exploited Long-Short Term Memory networks both to perform human activity recognition and to classify the weight of lifted objects up to 15 kg. We found a median F1 score of $90.80\%$ (activity recognition) and $87.14\%$ (payload estimation) with subject-specific models trained and tested on 12 (6M-6F) young healthy volunteers. We also succeeded in evaluating the applicability of this approach with an in-lab real-time test in a simulated target scenario. These high-level algorithms may be useful to fully exploit the potential of powered exoskeletons to achieve symbiotic human–robot interaction.

## Introduction

Exoskeletons are a wide-spreading technology with a dramatic potential to improve the quality of life for a large part of the world’s population. This is the case of low-back exoskeletons for the support of workers dealing with exhausting activities. Despite the revolution envisioned by Industry 4.0—that is the transition towards smart, autonomous factories—robots are not yet ready to replace humans in a dexterous workspace. Indeed, tasks may be characterized by either lack of repetitiveness, changing requirements, or both. As a consequence, the industrial sector is still among the ones with the highest work intensity, according to the sixth European Working Conditions Survey^1^. In particular, low-back pain (LBP) was the leading cause of disability in 2015 and it has been the leading cause of years lived with disability for several decades^2^. The impact of low-back pain was also found to be increasing ($+18.6\%$) in the decade 2005–2015^2^. Bending, lifting weights, and twisting were found to have the highest potential of inducing low-back pain^3^. In the case of occupational low-back pain, activities related to Manual Material Handling (MMH), and in particular tiring or painful postures and repetitive load lifting are among the main triggering factors. In particular, cumulative low-back loading was found to be a significant risk factor for low-back pain as firstly reported by Coenen and colleagues^4^.

Low-back exoskeletons have been developed to assist workers with the most tiring and stressful tasks. They are intended to reduce the compression forces and the resulting torques acting on the spine, especially at the level of the lumbo-sacral (L5/S1) joint. Recent works have investigated the effects of such assistive devices on the physical workload^5,6^, as well as the efficacy of the evaluation standards used to measure such effects^7^. Current solutions mostly consist of active (or powered, or robotic) and passive (or unpowered) exoskeletons. Active exoskeletons are typically battery powered and feature one motor per each actuated degree of freedom. They can provide higher bursts of power and tune the assistance to be more versatile. On the other hand, passive exoskeletons exploit elastic elements, such as springs. They provide assistance by releasing deformation energy previously stored as kinetic energy provided by the wearer. While being simpler, lighter, and cheaper, passive exoskeletons only have a single equilibrium point, that depends on the pre-load of their elastic elements. Hence, there is a trade-off between usability and assistance: since the energy they provide is (partly) provided by the human user, passive exoskeletons generally provide lighter assistance. Active exoskeletons can provide higher power and are designed with the potential to finely tune their output torque and thus provide adaptive assistance to the wearer, at the cost of being heavier, more expensive, and more complex.

Despite the promising potential of exoskeletons, their application in the industrial scenario has been a challenge so far^8^. Indeed, occupational exoskeletons still have a low technology-readiness level (TRL), and this is among the main factors limiting their real-life adoption^9^. Passive exoskeletons still outnumber active exoskeletons in terms of published evaluation studies^6^, and this may suggest that they are preferred in the industrial context. Among other factors, their higher reliability and lower cost may have boosted in-field tests and adoption. They also have greater user acceptability, as they function in an intuitive and repeatable way. On the other hand, their main limitation is the lack of versatility: they are designed and tuned to assist a single task. Moreover, recent literature is highlighting the presence of a technical gap between active and passive occupational exoskeletons for industrial use^6,10^, another factor that could explain the bias towards the passive technology in the industry. On the other hand, active exoskeletons should be further investigated to exploit their higher potential, as they could provide adaptive assistance and higher power. Filling this technological gap would potentially increase (active) low-back exoskeleton adoption, with the aim of reducing the impact of work-related musculoskeletal disorders thus improving the quality of life of industrial workers.

One strategy to exploit the superior potential of robotic exoskeletons would be through adaptive assistance. The exoskeleton should activate automatically when necessary, adapting the provided torque to the current task and the payload. This would allow the exoskeleton to effectively reduce the stress on the musculoskeletal system while being *transparent* to the wearer. This means that the goal is to avoid having any kind of user input (i.e., voice activation or manual triggers). This aim should be achieved without sacrificing end-user ergonomics and acceptability, not to increase the invasiveness of the exoskeleton by means of additional sensors, or the cognitive load required to use the device. In the case of occupational low-back exoskeletons, assistance adaptation should therefore rely on activity recognition and automatic assistance adaptation with respect to the manipulated payload.

## Related work and paper contribution

Pioneering active low-back exoskeletons for industrial use required user input to provide assistance. This could be easily obtained using control buttons. However, direct user input limits the usability of the device and increases the cognitive load on the end user. EMG-based control has been implemented because it allows both motion-intention detection and assistance modulation during the task^11^. Yet, electromyography is not often used because of several drawbacks. For instance, expertise in positioning the electrodes is required, and long-term use (as in an 8-hour working day) may be subject to decreasing signal-to-noise ratio because of changes at the skin-electrode interface. Sweating, for example, can reduce the amplitude of the electrical signal and introduce differential noise, thus lowering the reliability of EMG for long-term control applications.

In recent years, researchers in the field started to investigate control strategies for lift assistance relying only on sensors already integrated within low-back industrial exoskeletons. These often do not include force/torque sensors, traditionally embedded in each degree of freedom of a robotic device. Novel actuators that are being designed for industrial exoskeletons—such as series elastic actuators (SEA)^12^—often do not feature such expensive sensors. Chen et al.^13^ recently proposed an online algorithm for lift detection to be used in a SEA-based occupational exoskeleton. The algorithm is able to understand when the wearer is lifting and lowering a box. This simple-yet-effective control scheme exploits kinematic data measured on the exoskeleton (hip encoders) and on the wearer (trunk inertial sensor). Similarly, Poliero et al.^14^ developed a Support Vector Machine classifier able to distinguish several actions (walking, bending, standing) from wearable inertial sensors. This was aimed to improve the versatility of a low-back exoskeleton for industrial use by implementing Human Activity Recognition (HAR)^15^.

Payload estimation and compensation could be achieved by exploiting classical inverse dynamics^16^ or electromyography^17,18^, or a combination of the two. To achieve this, the exoskeleton needs to be equipped either with force/torque sensors or EMG electrodes, respectively, jeopardizing wearability, task-technology fit, and end-user willingness-to-use, already critical in the industrial context^19,20^. Indeed, workers’ perceived ease-of-use is the most important factor for predicting their intention-to-use^21^. To the best of our knowledge, there is no record in the literature of automatic payload compensation methods based on the use of inertial sensors. On the other hand, a method for non-collocated payload and wrench estimation for online human ergonomics assessment was recently published^22^. This method achieved good results by exploiting data from 17 wearable inertial sensors and sensorized shoes featuring 2 force/torque sensors each. Although accurate and robust, this method relies on a rather complex sensor configuration, affected by poor wearability, as pointed out also by the authors. EMG-based load classification algorithms have been recently developed. Totah et al.^17^ developed a multi-nomial logistic regression algorithm that could classify dynamic lifting tasks within three classes (0, 4.5 and $10.8\, \textrm{kg}$) achieving just above 80% accuracy. Higher accuracy was obtained by Aziz et al.^23^. In their work, a Support Vector Machine with cubic kernel fit on EMG data achieved 99% accuracy for the classification of $1\, \textrm{kg}$, $3\, \textrm{kg}$ and $7\, \textrm{kg}$ payloads.

In this work, we present a solution solely based on Inertial Measurement Units (IMU) that exploits deep learning for human activity recognition (HAR) and payload estimation (PE). In particular, we aim to obtain a payload classification algorithm using inertial data only, as IMU’s are easily wearable or embeddable in any industrial exoskeleton. Similarly, we exploit the same data to perform HAR, as done by a few similar approaches found in the literature. For this purpose, we exploited a Recurrent Neural Network (RNN) to obtain subject-specific models.

The end goal of this work is to develop automatic control systems for a low-back exoskeleton. These should allow achieving automatic payload compensation in real time: the exoskeleton should first recognize the interaction with an object, and then assist the wearer *as needed*, i.e., modulating the provided torque with respect to the estimated payload. To achieve our goal, we identified three major steps. First, we designed and validated deep-learning algorithms to perform HAR, detect object interaction, and finally estimate the payload by means of classification. Second, we investigated the minimum number of inertial sensors required to achieve adequate classification performance. Third, we evaluated the computational cost and the feasibility to implement such algorithms to achieve real-time predictions for automatic exoskeleton control. These steps are detailed in the remaining of this paper.

## Methods

### Experimental setup

The experimental setup consisted of five wireless Inertial Measurement Units (IMU). We used the Xsens MTw Awinda system (Xsens Technologies B.V., Enschede, NL) that features 9-axis inertial sensors. Each sensor is equipped with a 3-axis accelerometer, a 3-axis gyroscope, and a 3-axis magnetometer. We developed custom software based on the C++ SDK provided by the manufacturer to acquire 6-axis data (accelerometer and gyroscope) from each IMU. Data were sampled at 100 Hz and then processed offline. We *a priori* excluded the magnetometer for two reasons: to avoid dealing with calibration and interference issues and to reduce the dimensionality of the data. Indeed, we believe that the information provided by the magnetometer may be redundant in our case and thus not useful for the model.

The setup also included the manipulated payloads. We used a warehouse box (AUER Packaging, Amerang, DE) of size $40\times 30\times 22 \ \textrm{cm}^3$ and mass $1.3\ \textrm{kg}$ to store from one to three 5-$\textrm{kg}$ discs, thus achieving payloads of $5\ \textrm{kg}$, $10\ \textrm{kg}$, and $15\ \textrm{kg}$.

We developed custom software running on a laptop both to communicate with the Xsens Awinda station and to provide visual feedback to the subjects during the data acquisition protocol.

**Figure 1:**
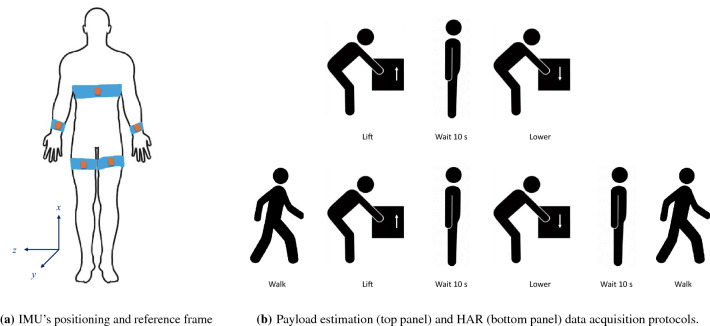
**(a)** IMU positioning, **(b)** experimental protocols.

### Data acquisition protocols

We enrolled 12 sex-balanced (6M-6F) young healthy volunteers (height $1.76 \pm 0.1\ \textrm{m}$, weight $60.7 \pm 10.7\ \textrm{kg}$, age $25.1 \pm 1.2$ years) to participate in the experimental data acquisition protocol, upon approval of the Ethical Committee of the Politecnico di Milano (Opinion no. 13/2021). Ouf of the 12 test subjects, only 1 had work experience with MMH tasks, while the rest had never been involved or employed in this field at the time of the study. The experimental campaign was conducted following the Declaration of Helsinki and in respect of the in-force directives for the COVID-19 pandemic. All subjects provided written informed consent, including consent to publish images or videos taken during the experimental protocol. The overall data acquisition process—including donning and doffing the sensors—lasted approximately 30 minutes per subject.

Each subject was instructed to wear the five wireless IMU’s with the help of elastic bands, under the supervision of one researcher. The first IMU was placed on the chest, at the sternum level. The other four were placed bilaterally on the wrists and on the thighs. All IMU’s were aligned with their *x*-axis parallel to the longitudinal direction of the corresponding body segment pointing upwards in the standing position. Sensor positioning is shown in Fig. 1a, where we also show the orientation of the IMU’s reference frame.

We designed two experimental protocols to acquire training data for our deep-learning models. Specifically, we need to have a dataset for each model to perform Human Activity Recognition (HAR) and Payload Estimation. The two protocols consist of typical actions for industrial workers. The first protocol is specifically designed for payload estimation. It only includes the payload manipulation actions, as shown in Fig. 1b (top panel). In particular, subjects lifted (and released on a desk) and lowered (and positioned onto the ground) a box while standing in front of a desk, with a 10-s pause in between each action. Subjects completed 10 repetitions of the material manipulation cycle with three different payloads: 5, 10, and $15\ \textrm{kg}$ (i.e., 3 sets of 10 repetitions for a total of 30 lifting repetitions and 30 lowering repetitions). The first experimental data acquisition gives the first dataset (D1) for model training. The second protocol was designed for HAR. Specifically, it includes activities typical of the end-user scenario, such as standing still, walking, and payload manipulation, as shown in Fig. 1b (bottom panel). With this protocol, we acquired two datasets. First, we ask subjects to perform 10 repetitions with a fixed payload value of $10\ \textrm{kg}$: in this way, we obtained the second dataset (D2). Then, we repeated the HAR protocol counting two repetitions for each payload value (i.e., 3 sets of 2 repetitions) to acquire dataset D3. The combinations of experimental protocols and payloads that results in the three datasets are shown in Table 1. Subjects were asked to perform the movements as naturally as possible, in order to achieve real-life inter-repetition similarity.

**Table 1:** Datasets used for model training and testing obtained combining data acquired with the experimental protocols designed for this study.

Dataset	Experimental protocol	Payload (kg)	Repetitions	HAR model	PE model
D1	Protocol 1	5, 10, 15	$10 \times 3$	–	90–10% train-test
D2	Protocol 2	10	10	100% train	–
D3	Protocol 2	5, 10, 15	$2 \times 3$	100% test	50–50% train-test

### Deep learning framework

Input-output pairs are required to train a model following the concept of supervised learning. In our case, the input dataset (*U*) is the collection of the inertial data measured by the five IMU’s worn by each subject. Specifically, we have three linear acceleration signals and three rotational velocity signals for each sensor, for a total of 30 input features. The target variable (*y*) is the action for the HAR model and the manipulated payload ($\textrm{kg}$) for the payload estimation model. For each dataset, labeling was done semi-automatically (also exploiting data from a sixth IMU attached to the box) and manually adjusted if necessary.

#### Data pre-processing

Raw 6-axis data acquired from each of the five IMU’s were stored for offline processing and model training. As a requirement, we limited the pre-processing steps in order to obtain results that are easily reproducible online with limited computational power. All data pre-processing was done using Python (version 3.9.6).

First, we low-pass filtered each signal at 5 Hz using a zero-lag, fourth-order Butterworth filter. The cut-off frequency of the filter is set according to the typical bandwidth of human body motion^24^. Then, we standardized the input data by means of scaling the amplitude of each continuous feature between $-1$ and $+1$. Specifically, we fit a data transformer (MinMaxScaler) for each model on its training data only and applied the resulting transformation to the test datasets in the model evaluation phase. The pre-processing steps are minimized in order to be applied online with limited computational power.

#### Network architecture and training

The deep learning framework for this project is based on the open-source libraries *scikit-learn*^25^ and *TensorFlow*^26^ for Python. We opted for a recurrent neural network (RNN) architecture to solve a multi-class classification problem with time series. In particular, we chose the Long Short-Term Memory (LSTM)^27^ layer to implement the recurrent layer(s) of our deep-learning network. Feedback connections typical of this architecture allow it to be well suited to classify input sequences.

**Figure 2:**
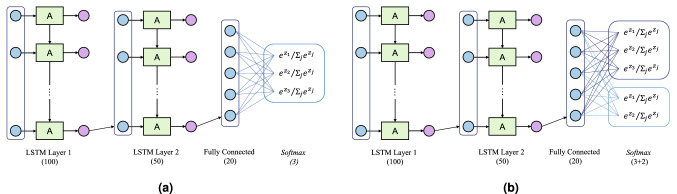
Network architecture featuring LSTM and fully-connected layers: **(a)** payload estimation and **(b)** multi-output network for HAR.

We have designed a similar network architecture to tackle both HAR and payload estimation. In particular, this consists of a series of Long-Short Term Memory (LSTM) layers and conventional fully-connected layers. The former are recurrent neural network (RNN) layers that provide memory to the network. The LSTM is the state-of-the-art approach for time series classification^28^. Finally, the output layer is a *P*-neuron fully-connected layer with *softmax* activation function, to provide the probability for each class $p_i$ (where *P* is the number of total classes). For payload estimation ($P=3$), we defined a model with two LSTM layers followed by a single fully-connected layer and an output layer. These layers feature 100, 50, and 20 neurons, respectively, while the output layer has 3 units. The architecture of this model is shown in Fig. 2a. For HAR, we exploited the same architecture, this time to define a multi-output model. In particular, we split activity recognition into two tasks: first, we want to distinguish among standing still, walking, and interaction with the payload. Then, we want to further distinguish between lifting and lowering the payload. In the following, we refer to this as *action* and *interaction* prediction, respectively. This model thus features two output layers, with 3 and 2 neurons for action and interaction-type recognition, respectively. This architecture is shown in Fig. 2b. The multi-output model allows reducing the overall complexity of the system, as a single network needs to be trained and deployed to production, also limiting training time and run-time computational requirements.

**Figure 3:**
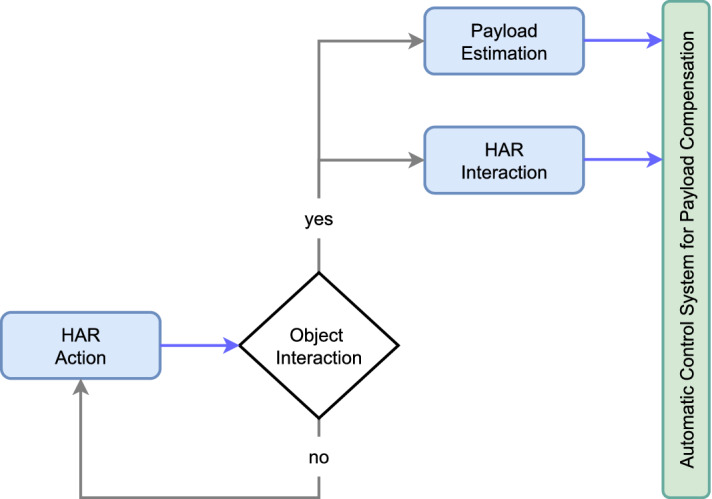
Flowchart of our deep learning models for exoskeleton control: interaction type (HAR) and payload estimation are triggered only when the predicted action (HAR) is equal to object interaction. In the figure, blue arrows represent model predictions, grey arrows represent flow actions.

The training datasets for the two models are defined differently according to the different data acquisition protocols described above. Specifically, for the HAR model, we used dataset D2 for training and D3 for testing (i.e., evaluating the model performance on *unseen* data). On the other hand, we trained the payload estimation model using 90% of dataset D1 and 50% of D3; the other half of D3 is used for testing, together with the remaining 10% of D1. For the payload model, D3 is split in half to provide additional training samples. Testing is done on both protocols to better evaluate the performance of the model. Train-test splits of the datasets for each model are shown in Table 1. The test set is acquired in order to mimic the real-world deployment of both models. In particular, we evaluated both models on the HAR protocol (see Fig. 1b, bottom panel) with all payload values. Please note that in this case data is fed to the payload estimation model only when the HAR model predicts object manipulation, as in a real-world application. Indeed, our models are *interconnected* in a control-logic approach, as shown in Fig. 3.

We trained subject-specific models to allow for inter-subject variability of task kinematics, expected to be large.

#### Sensor reduction

We evaluated sensor reduction as a *grey-box* way to reduce the dimensionality of the input data. Indeed, black-box methods would potentially include a sub-set of features for each IMU, instead of applying a sensor reduction approach. Instead, here we are interested in finding the minimum number of sensors and their optimal configuration that still allows achieving good classification accuracy compared to the full configuration. In particular, we aim to find the best configuration in terms of user comfort and acceptability. Sensor reduction was done in two steps. First, removing the data acquired by the sensors worn on the wrists. Indeed, these sensors could not be directly embedded into a low-back exoskeleton and would require additional donning/doffing steps, potentially jeopardizing end-user’s intention to use. Then, we kept only the data measured by the IMU on the chest. The reduced-size datasets count 18 features (40% dimensionality reduction) and 6 features (80% dimensionality reduction), respectively. On the reduced datasets, we applied the same DL framework for classification, re-training the models and then evaluating their performance, as detailed below.

In order to understand whether any difference in performance among the three configurations would be statistically significant, we performed either the paired-samples T-test (for normal data distributions) or the Wilcoxon signed-rank test (non-parametric method for non-normal data distributions). Normality was tested with the Shapiro–Wilk test ($\alpha = 0.05$). Specifically, we compared each configuration against another in terms of F1 score. Then, we compared the resulting p-values with the significance threshold obtained applying the Bonferroni method, i.e., $\alpha = 0.05/3 = 0.0167$. Corrected p-values are reported with the symbol $p^*$ (such that $p^* =3\cdot p$). All tests were performed using Python v3.9.6 and the open-source library Scipy v1.9.0.

#### Performance evaluation

We used the categorical cross entropy as our loss function and the weighted F1 score to evaluate different models during the training phase. This score (Eq. 1) is defined as the harmonic mean of precision ($Pr$) (Eq. 2) and recall ($Re$) (Eq. 3). These quantities depend on the number of true positives ($TP$), false positives ($FP$), true negatives ($TN$), and false negatives ($FN$). Finally, we can compute the accuracy (Eq. 4) of the model as the ratio between the correct predictions and the total number of predictions.

1 $$\begin{aligned} F_1&= 2 \cdot \frac{ Pr \cdot Re }{ Pr + Re } \end{aligned}$$

2 $$\begin{aligned} Pr&= \frac{ TP }{ TP + FP } \end{aligned}$$

3 $$\begin{aligned} Re&= \frac{ TP }{ TP + FN } \end{aligned}$$

4 $$\begin{aligned} Ac&= \frac{ TP + TN }{ TP + TN + FP + FN } \end{aligned}$$

As we deal with a multi-class classification problem, precision, recall, and F1 score are computed for each class and then averaged to obtain a single score. Finally, we evaluated the distribution of each score across all subjects.

As mentioned throughout this manuscript, we want to obtain models not only able to perform HAR and payload estimation accurately, but also capable of doing so *online*. Thus, we evaluated the model both in terms of prediction speed and accuracy. We exploited the TensorFlow Lite library^29^ to deploy our model for online testing. For online testing, IMU data was sampled at 100 Hz (as for offline training), single-pass filtered online with the aid of a circular buffer, then scaled between $-1$ and $+1$ with the scaler trained offline, and finally fed to the models.

## Results

### Classification performance

The performance of the LSTM-based classifier are shown in Table 2 for the multi-output HAR model and in Table 3 for payload estimation. Results are hereby reported as first quartile, median, and third quartile across the study population ($N=12$), as their distributions were not normal.

**Figure 4:**
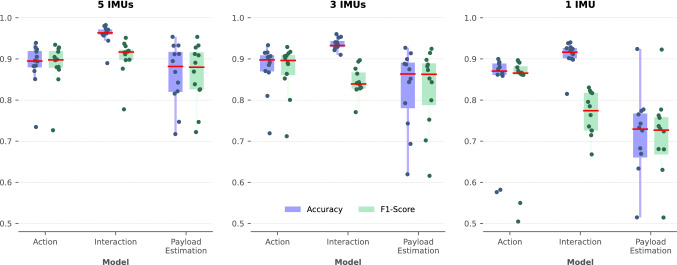
Boxplot-like plot of accuracy (blue) and F1-score (green) showing median value (red line), individual subject scores (dots), and quantile-based ranges (colored boxes). Results are shown for full sensor configuration (left panel) and reduced configurations (middle and right panels).

In terms of accuracy, the HAR model achieved a median value of $89.49\%$ for *action* prediction (stand/walk/payload-interaction) and $96.33\%$ for the *interaction type* prediction (lifting/lowering). The payload estimation model achieved a median accuracy value equal to $88.16\%$. For real-world classification problems, high accuracy must be matched by high F1-score values, which guarantee the model can also work with unbalanced datasets. In our case, we found median values of $89.92\%$, $91.68\%$, and $87.14\%$ for the three models. Accuracy and F1-score values are also shown in the boxplot charts of Fig. 4 (leftmost panel). Prediction errors were found to be crowded in the transitions of the true label (i.e., change of payload value or change of action) and, in the case of the payload model, during the second half of the lifting movement.

**Table 2:** Results of multi-output HAR model (full sensor configuration): first quartile, median value (bold font), and third quartile shown for both outputs.

	Action				Interaction		
$F_1$ Score	0.8697	**0.8992**	0.9208		0.8976	**0.9168**	0.9238
Recall	0.8778	**0.9037**	0.9400		0.9084	**0.9352**	0.9603
Precision	0.8647	**0.9032**	0.9309		0.8719	**0.8897**	0.9344
Accuracy	0.8794	**0.8949**	0.9190		0.9592	**0.9633**	0.9714

**Table 3:** Results of payload estimation (full sensor configuration): first quartile, median value (bold font), and third quartile.

**Payload estimation**			
$F_1$ Score	0.8194	**0.8714**	0.9173
Recall	0.8030	**0.8908**	0.9264
Precision	0.8013	**0.8986**	0.9413
Accuracy	0.8199	**0.8816**	0.9170

### Sensor reduction

Sensor reduction was found to be a rather effective way to implement dimensionality reduction. Specifically, we compared the models trained with the full sensor configuration (5 IMUs) with two *reduced* configurations, namely considering only 3 (chest and legs) and 1 (chest) inertial sensor.

The performance of the multi-output model for HAR is almost unaffected by the first sensor reduction step (i.e., comparing the 3-IMU configuration with the full configuration). Specifically, the median accuracy and F1 score are equal to $89.77\%$ ($86.97 - 90.88\%$) and $89.49\%$ ($86.41 - 91.23\%$), respectively. The same can be said for the second output of this model (i.e., the interaction type prediction) although there is a greater difference in performance compared to the full sensor configuration: the median accuracy and F1 score are equal to $93.26\%$ ($92.87 - 94.34\%$) and $83.94\%$ ($82.92 - 86.71\%$), respectively.

Rather interestingly, the single-sensor configuration maintained adequate performance. Indeed, the multi-output model metrics were equal to $87.02\%$ ($86.09 - 88.87\%$) and $87.17\%$ ($84.03 - 89.05\%$), respectively for accuracy and F1 score, for the action prediction, and equal to $91.63\%$ ($90.15 - 92.64\%$) and $77.13\%$ ($72.62 - 81.90\%$) in the case of the lifting/lowering prediction. The interaction-type prediction was affected the most by sensor reduction.

For the action prediction of the multi-output HAR model, only the difference between the 5-IMU and 1-IMU configurations resulted statistically significant ($$p^*=0.0103$$), while it was not significant for the other two comparisons ($$p^*=0.4541$$ and $$p^*=0.1919$$, respectively). On the other hand, the interaction-type prediction of the same model was significantly affected by sensor reduction. Specifically, all differences between configurations were found to be statistically significant: the 5-IMU configuration is the best both when compared to the 3-IMU ($$p^*=0.0103)$$ and to the 1-IMU configuration ($$p^*=0.0015)$$, while the 3-IMU configuration is better than the single IMU configuration ($$p^*=0.0016)$$.

The payload estimation model showed similar behavior, although with larger performance drops. Specifically, the median accuracy was equal to $86.34\%$ ($78.01 - 89.10\%$) and $72.93\%$ ($66.03 - 76.73\%$) for the 3-IMU and 1-IMU configuration, respectively. Similarly, the median F1 score was equal to $85.06\%$ ($78.20 - 89.41\%$) and $70.92\%$ ($65.12 - 76.96\%$).

Each configuration was found to be significantly different from the others. Again, the full sensor configuration is better both than the 3-IMU ($$p^*=0.0366)$$ and 1-IMU configurations ($$p^*=0.0015)$$, and the 3-IMU configuration is better than the 1-IMU configuration ($$p^*=0.0015)$$.

Figure 4 shows accuracy and F1 score for the two models (multi-output HAR model and payload estimation model) both for the full sensor configuration (left) and for the reduced configurations (middle and right panels).

### Time-domain performance

We evaluated the time-domain performance of our classifiers with an online test. Both models were able to correctly classify the action and the interaction—HAR model—as well as the payload value. Although performed on a single subject, this test is crucial to guarantee the feasibility of running such models online while keeping their high accuracy after offline training and testing. This test is shown in Supplementary Video 1 available online. The video shows the test subject performing the actions while the real-time prediction of the models is shown on screen at the prediction rate (100 Hz).

With the aim of embedding our models into the high-level control system of a low-back exoskeleton, we also evaluated their computational cost. We measured an average inference time equal to $1.07\, \pm \, 0.08\ \textrm{ms}$ and $1.93\, \pm \, 0.49\ \textrm{ms}$ on a general purpose processor (Intel i7-10510U, 1.80 GHz) for the multi-output HAR model and payload estimation model, respectively. The prediction update rate is actually limited by the sampling frequency of our IMU system (Xsens Awinda). Furthermore, we exploited TensorFlow Lite to derive a lighter model for embedded systems and successfully ran inference on the Teensy 4.1 microcontroller (ARM Cortex-M7, 600 MHz) streaming IMU data to it over a serial connection. Both solutions are adequate for running both models at the full data sampling rate (100 Hz).

## Discussion

With this work, we propose the use of deep learning for human activity recognition and payload estimation based on wearable inertial sensors. We obtained subject-specific classifiers that we aim to deploy online in a low-back exoskeleton. In this Section, we point out the main findings of this study, focusing on the raw performance of the models and their applicability.

Starting with the payload estimation model, the LSTM-based architecture reached a rather high and robust performance level. The issue of inter-subject variability during MMH tasks—resulting in different kinematics across subjects—was solved by training subject-specific models. We believe this is fundamental for real-world applications in the field of industrial exoskeletons, as it avoids adding constraints to the end users. Indeed, it allows subjects to perform the *material manipulation cycle* as naturally as possible. This means not forcing squatting or stooping. In this way, the model can *learn* the style of each subject, and thus it can achieve the same level of accuracy across different subjects. On the other hand, subject-specific models are inevitably trained with low-dimensionality datasets. This means that high classification accuracy was reached with relatively small datasets, only made of 10 repetitions for each payload value per subject. Transfer learning was not implemented in this study as it was found to be not effective in a preliminary analysis. A pre-trained model—i.e., trained with all the data and finely tuned for each subject—would probably require either hundreds of subjects or more model parameters, or both, thus increasing the computational cost. Indeed, we achieved these results with limited pre-processing and network complexity, avoiding the computation of complex features from raw IMU data and limiting the *depth* of the network. Another advantage of our approach is that it does not need to generalize to different payload values or test populations. Being the models specific to each subject, they can be trained adjusting the number of classes and the payload values while adapting to each subject lifting technique by design. In principle, this will guarantee similar performance when tested and deployed on real end users.

It is difficult to directly compare the efficacy of our method with respect to similar EMG-less methods for payload estimation or compensation. We mentioned the work of Toxiri et al.^18^, in which the authors implemented an adaptive payload compensation strategy based on electromyography. Specifically, the torque provided by their exoskeleton was set to be proportional to muscular activity, measured while lifting two different payloads ($7.5\ \textrm{kg}$ and $15\ \textrm{kg}$). Tirupachuri et al.^22^ achieved sub-kilogram resolution with their payload estimation method, relying on a rather broad and complex sensor configuration. Their inverse-dynamics approach requires measuring the ground reaction force and the motion of the whole body with 17 inertial sensors. In contrast, in this work, we set user comfort and ease of use as a priority in determining the sensor configuration. Moreover, our LSTM model achieved a level of accuracy comparable to state-of-the-art EMG-based methods, as shown in Table 4. Although electromyography could reach higher accuracy and potentially better control-oriented performance thanks to the electromechanical delay, our method falls between the two with a much more user-friendly and *reliable* sensor configuration and was tested with a wider payload range.

**Table 4:** Performance comparison of our method (*) and state-of-the-art EMG-based approaches.

Model	Method	Sensors	Payload values (kg)	Accuracy (%)
Multi-nomial logistic regression^17^	Classification	EMG	0, 4.5, 10.8	81.00
Cubic SVM^23^	Classification	EMG	1, 3, 7	99.00
LSTM*	Classification	IMU	5, 10, 15	88.16

The LSTM-based network achieved satisfying results for human activity recognition as well. The multi-output model reached a robust performance level both for action and interaction-type predictions.

Our models rely solely on inertial sensors. IMU’s are cheap and easy-to-integrate sensors that could either be worn by the subject or attached to the frame of the exoskeleton. Moreover, they are preferable to other sensors that could potentially be used for this purpose, such as sensorized gloves or electromyography electrodes. IMU’s are more robust to different grasping techniques—that may depend on the shape of the object—and do not require direct skin contact. This provides higher usability and hence fewer constraints for the end user.

We applied sensor reduction as a *grey-box* dimensionality reduction technique. We have seen that the 3-IMU sensor configuration (chest and legs) may be the optimal solution to the trade-off between prediction accuracy and overall user experience, improving the wearability and also reducing costs and encumbrance of the setup. This configuration is suitable for low-back exoskeletons, as the chest IMU could be positioned either on the soft human-exoskeleton interface or into the rigid structure of the back, and the two sensors on the thighs could be embedded in the soft interface that unloads the device below the hips. A single IMU on the chest may be used in highly-constrained scenarios while still maintaining good performance, especially in the case of HAR. This sensor reduction analysis also allows us to gather some insights into the functioning of our black-box deep-learning models. The kinematics of the torso is clearly the most important source of information for the models, being sufficient for a good *first guess* of the prediction. This is shown comparing the performance of the 1-IMU configuration with the full-sensor configuration (cfr. Fig. 4). When more data is available, the model can further exploit legs and arms kinematics to improve its ability to distinguish different payload values, or the interaction with the object, with higher accuracy. The IMUs on the wrists only provide a fractional increase of performance to the payload model and even slightly decrease the metrics for HAR. These sensors could be safely removed to reduce the complexity of the system, or re-positioned in an attempt to improve performance and robustness.

The main limitation of our work regards payload estimation. Our models do not provide a direct weight estimate: they can classify different payloads seen during training based on inertial sensor data. This *indirect* payload estimate can be exploited to *classify* lifted payloads instead of giving the exact weight of the object being manipulated. In our vision, payload classification based on inertial data could be exploited to achieve adaptive assistance by means of a step-wise gain-scheduling approach. Specifically, this information could be used to tune the assistance of the exoskeleton for different payload values. Fine resolution of the payload estimate may be not required for occupational exoskeletons, as they generally do not provide $100\%$ compensation of external loads. For this reason, we opted for classifying the manipulated payload in several classes (i.e., 5, 10, and $15\, \textrm{kg}$). We found it is possible to solve such a classification problem with quite a high accuracy. We also investigated sensor reduction and showed how a single IMU on the chest could be used to rather accurately perform activity recognition and classify the lifted payload.

## Conclusion

Industrial low-back exoskeletons have great potential in reducing the impact of work-related musculoskeletal disorders in a high-intensity working environment. Yet, their adoption rate is still below expectations. One reason could be the lack of adaptive control algorithms able to tune the assistance. In this respect, we believe that industrial robotic exoskeletons could benefit from cost-effective human-activity recognition and payload estimation algorithms.

In this work, we propose a deep-learning approach for Human Activity Recognition (HAR) and payload estimation based on inertial sensors. We developed a multi-output model that can distinguish among standing still, walking, and object interaction (lifting/lowering), and a payload estimation model that can classify weights of 5, 10, and 15 kg. The presented classifiers were tested on 12 healthy volunteers revealing satisfying performance both in terms of evaluation metrics and online applicability. Nevertheless, there are some limitations that are worth pointing out. The most relevant is probably related to target generation for supervised learning, which could impact the results. As mentioned above, prediction errors—especially for the payload model—are crowded either in the transitions of the output (when grasping or releasing the payload) or in the second half of the movement. This is quite evident in the Supplementary Video 1. For the classification of a time series, how transitions among actions (i.e., classes) are handled is still an open problem. When labeling the dataset, either automatically (e.g., by means of user-defined thresholds) or manually, the choice is quite arbitrary. While the transition between two classes can last hundreds of milliseconds, the true label changes in a single time step (i.e, $10 \textrm{ms}$). In this work, we decided to include the transition phase into the subsequent class. For example, the transition from standing still to object interaction (i.e., lifting or lowering) is labeled as the latter. Indeed, we want our classifiers to *identify* actions as soon as they are started. Both the action performed and the payload value are estimated by the classifier in the initial instants of lifting, so that the exoskeleton can tune the assistance with the correct timing. After this, model predictions are ignored by the exoskeleton controller, and thus their accuracy in the second half of the action is less relevant for our application. In the trade-off between promptness and accuracy, when labeling our data we opted to optimize the former.

### Future work

Adaptive payload compensation could dramatically improve the user experience of low-back exoskeletons, reducing the impact of low-back pain in the industrial sector. The next step of this project would be embedding the classifier into the control system of a low-back exoskeleton and testing its performance in terms of assistance to the wearer. We plan to do such tests in the near future with a prototype under development. Embedding the IMU’s into the exoskeleton will allow for repeatable sensor positioning while potentially changing the kinematic patterns measured during the tasks. It will be challenging also to see how the classifiers will perform with different payload values and lifting techniques. Another challenge will regard the real-time performance of the classifiers: increasing the IMU sampling frequency, tuning the architecture of the LSTM-based models or re-training them online could be investigated in order to overcome the limitations seen during the online test of the models.

## Supplementary Information

Supplementary Video 1.

## Data Availability

The datasets that support the findings of this study are published online on Zenodo, and accessible at the following link: https://zenodo.org/record/7182799.
